# Dengue Infection in Children in Fortaleza, Brazil: A 3-Year School-Based Prospective Cohort Study

**DOI:** 10.4269/ajtmh.19-0521

**Published:** 2020-04-27

**Authors:** Ivo C. B. Coelho, François Haguinet, Jeová Keny B. Colares, Zirlane C. B. Coelho, Fernanda M. C. Araújo, Waleska Dias Schwarcz, Ana Claudia Duarte, Beatriz Borges, Catherine Minguet, Adrienne Guignard

**Affiliations:** 1 Universidade Federal do Ceará, Fortaleza, Brazil;; 2 GlaxoSmithKline, Wavre, Belgium;; 3 Secretaria de Saúde do Estado do Ceará, Hospital São José de Doenças Infecciosas, Fortaleza, Brazil;; 4 Programa de Pós-Graduação em Ciências Médicas, Universidade de Fortaleza, Fortaleza, Brazil;; 5 Laboratório Central de Saúde Publica do Ceará, Fortaleza, Brazil;; 6 Bio-Manguinhos-Fiocruz, Rio de Janeiro, Brazil;; 7 GSK, Rixensart, Belgium

## Abstract

Dengue is endemic in Brazil. The dengue surveillance system’s reliance on passive reporting may underestimate disease incidence and cannot detect asymptomatic/pauci-symptomatic cases. In this 3-year prospective cohort study (NCT01391819) in 5- to 13-year-old children from nine schools in Fortaleza (*N* = 2,117), we assessed dengue virus (DENV) infection seroprevalence by IgG indirect ELISA at yearly visits and disease incidence through active and enhanced passive surveillance. Real-time quantitative polymerase chain reaction (RT-qPCR) and DENV IgM/IgG capture ELISA were used for diagnosis. We further characterized confirmed and probable cases with a plaque reduction neutralization test. At enrollment, 54.1% (95% CI: 46.6, 61.4) of children were DENV IgG positive. The annual incidence of laboratory-confirmed symptomatic dengue cases was 11.0 (95% CI: 7.3, 14.7), 18.1 (10.4, 25.7), and 10.2 (0.7, 19.7), and of laboratory-confirmed or probable dengue cases with neutralizing antibody profile evocative of dengue exposure was 13.2 (6.6, 19.9), 18.7 (5.3, 32.2), and 8.4 (2.4, 19.2) per 1,000 child-years in 2012, 2013, and 2014, respectively. By RT-qPCR, we identified 14 DENV-4 cases in 2012–2013 and seven DENV-1 cases in 2014. During the course of the study, 32.8% of dengue-naive children experienced a primary infection. Primary inapparent dengue infection was detected in 20.3% (95% CI: 13.6, 29.1) of dengue-naive children in 2012, 8.7% (6.9, 10.9) in 2013, and 5.1% (4.4, 6.0) in 2014. Our results confirmed the high dengue endemicity in Fortaleza, with active and enhanced passive surveillance detecting three to five times more cases than the National System of Disease Notification.

## INTRODUCTION

Half of the world’s population lives in regions at risk of dengue infections.^1,2^ Dengue is mostly encountered in tropical and subtropical countries.^1^ In these regions, urban and suburban areas are particularly favorable for the spread of the *Aedes* mosquitoes, the vector of the dengue virus (DENV). In 2010, an estimated 390 million dengue infections occurred worldwide.^3^ Of these, 96 million manifested apparently with any level of disease severity, going from fever with mild nonspecific symptoms to dengue hemorrhagic fever and shock syndrome.^3,4^ The Americas bore 14% of the apparent dengue cases, half of them being recorded in Brazil and Mexico.^3^ In Brazil, dengue disease is endemic, and the number of yearly reported cases ranged from 565,510 to 1,649,008 during 2011–2016.^5^ Monthly dengue-related hospitalization rates increased between 1988 and 2015 in Brazil, especially in children.^6^ In 2019, 2.2 million cases were reported, representing a 10-fold increase compared with 2018.^7^

The high burden of dengue disease, the absence of specific treatment other than supportive measures,^8^ and the limited effectiveness of existing disease prevention methods, based on mosquito control and personal protection, highlight the need for an effective vaccine.^9^ A chimeric tetravalent dengue vaccine (Dengvaxia, Sanofi Pasteur, Marcy-l’Etoile, France) was licensed in several endemic countries across Asia and Latin America as of 2015. Its administration is only recommended for individuals previously infected with dengue, as clinical trials showed that the vaccine was efficacious and safe in previously infected individuals but carried an increased risk of severe dengue in those who experienced their first natural dengue infection after vaccination.^10^ In most countries, the age indication is 9–45 years. Still, for countries considering implementing a vaccination program, the WHO recommends a pre-vaccination serostatus screening program.^11^

In Brazil, the endemicity of dengue and the recent circulation of other arboviruses also transmitted by *Aedes aegypti*, such as Zika and chikungunya, reinforce the importance of a thorough characterization of arboviral disease epidemiology.^12–14^ The Brazilian dengue surveillance system relies on passive reporting from healthcare facilities (outpatient and hospital),^15^ with laboratory diagnosis for case identification and ascertainment of circulating DENV serotypes in a subset of suspected cases.^16^ This surveillance method may substantially underestimate dengue incidence because of the underreporting of symptomatic dengue cases.^17–19^ For instance, a previous study showed that only 50.7% of dengue hospitalizations were captured in the national surveillance system in Brazil.^15^ A study conducted in a public health emergency unit in Salvador, Brazil, between 2009 and 2011, estimated that overall there were 12 dengue cases per reported case in the community and > 17 dengue cases per reported case in months of low dengue transmission.^17^ Moreover, passive surveillance systems only provide a partial estimate of the burden of dengue because they do not detect inapparent infections, which represent most cases and contribute to disease transmission.^20,21^ A previous study conducted in Nicaragua showed that approximately six times as many dengue infections as symptomatic cases occurred in children.^22^

At the time of our study, there was a lack of characterization of the pediatric burden of dengue disease in Brazil in prospective cohort studies. To fill this knowledge gap, we evaluated the prevalence of past dengue infection and the incidence of symptomatic (primary and secondary objectives) and inapparent (secondary objective) primary dengue infections in 5- to 13-year-old children in Fortaleza over a 3-year period through active surveillance combined with enhanced passive surveillance. We also estimated the distribution of symptomatic dengue infection by each DENV serotype (DENV-1, DENV-2, DENV-3, and DENV-4). Finally, we also described the symptoms of dengue disease, and we evaluated the impact of disease in terms of school absenteeism and work absenteeism for the caregivers. The protocol is available at http://www.gsk-clinicalstudyregister.com (ID112994). Here, we report the results of all primary and secondary objectives and of one of the four exploratory objectives included in the protocol.

## METHODS

### Study design.

This school-based, prospective, cohort study involved approximately 2,000 children from nine schools in Fortaleza, Northeast Brazil, who were aged 5–13 years at the time of enrollment. The full study period lasted from September 2011 to January 2015 inclusive. We extended the initially planned study period of 2 years by 1 year to cover an additional dengue season. The first enrollment wave was performed between September 2011 and January 2012. Because of an issue with the verification of the status of the legally authorized representatives (LARs) of the children, enrollment was put on hold in January 2012 and took over in May and June 2012 (second wave of enrollment). The third and the last enrollment wave was performed between April 2013 and June 2013 to achieve the enrollment target.

**Figure 1.**
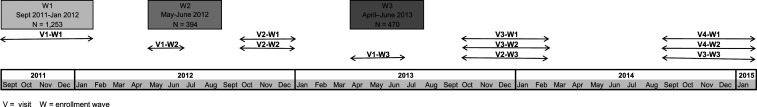
Timing of scheduled visits.

The children attended up to four scheduled visits. These visits were planned approximately 1 year apart, although it turned out not feasible to strictly respect these intervals for some participants (see the Results section and Figure 1). At the first visit, sociodemographic information, medical history, and yellow fever vaccination history were assessed. Physical examination was performed and a blood sample was collected to detect dengue infections that had occurred before enrollment with an indirect immunoglobulin (Ig) G enzyme-linked immunosorbent assay (ELISA). At each subsequent scheduled study visit (visits 2–4), physical examination was performed, medical history was updated, and a blood sample was collected for the assessment of dengue seroprevalence, to identify potential seroconversions between visits. The same indirect DENV IgG ELISA was used to detect past infections at each scheduled study visit.

Suspected dengue cases (SDCs) were detected through active school surveillance (tracking absenteeism caused by febrile illness) and “enhanced” passive surveillance outside school (i.e., parents/LARs and children of assent age were instructed on the signs and symptoms of dengue, how to take body temperature, and to contact the study team in case of febrile illness). In case of febrile illness at any time during the study, a medical appointment was scheduled at one of the two study sites. During this visit, the physician evaluated whether the child met the case definition for a SDC (fever on two consecutive days without an obvious reason unrelated to dengue). If the physician ruled out dengue suspicion (other obvious diagnosis), no further study procedures were conducted. For SDCs, the physicians collected information on the symptoms. Blood samples were collected during the visit for dengue diagnosis (acute sample), as well as for complete blood count and hepatic enzymes assessment. DENV IgM/capture IgG ELISA and real-time quantitative polymerase chain reaction (RT-qPCR) were used for laboratory confirmation. If the child did not require hospitalization, the parents/LARs received a diary card to record on a daily basis the clinical signs within 8 days following the initial visit (days 0–7). Any new outpatient visit (return visit) or hospitalization was recorded. A follow-up visit was scheduled 21–35 days following the initial visit. At this visit, the outcome of the illness episode was recorded and its overall severity was assessed. The impact of the episode in terms of absenteeism from school and absence of the parents/LARs from work was collected. A blood sample for dengue serology (IgM/capture IgG) was collected during the follow-up visit (convalescent).

All parents/LARs provided written informed consent, and children provided informed assent if applicable, in accordance with the International Conference on Harmonization Tripartite Guidelines for Good Clinical Practice. A local ethical committee in Fortaleza and the National Committee for Ethics in Research reviewed and approved the informed consent and assent forms and the study protocol. The study was conducted in accordance with the Declaration of Helsinki, Good Pharmacoepidemiology Practices,^23^ and all applicable regulatory requirements. The study is registered at http://www.clinicaltrials.gov (NCT01391819). Anonymized individual participant data and study documents can be requested for further research from www.clinicalstudydatarequest.com.

### Study population.

According to the Brazilian Institute of Geography and Statistics, Fortaleza was the fifth most populous state capital in Brazil in 2010, with approximately 2,315,116 inhabitants.^24^ This study included male and female children aged 5–13 years at the time of enrollment, who planned to attend one of the study schools for 2 years following enrollment. Children were eligible if, in the opinion of the investigator, they and/or their parent(s)/LAR(s) would comply with the requirements of the protocol. We excluded children planning to move away from the study area, children in care, or those enrolled in another study that would conflict with the current one.

The target sample size for this study was estimated based on local dengue epidemiology. In Fortaleza, in this age-group, the Ministry of Health reported an average incidence of symptomatic dengue of 0.35% between 2001 and 2006. To account for the underreporting, three different assumptions were considered: the true dengue incidence was similar to or was 5 or 10 times higher than the incidence reported by the Ministry of Health, that is, 0.35%, 1.73%, or 3.46%. The second scenario was considered to be the most likely. Assuming a lost to follow-up rate of 10% per year, a sample size of 2,000 children aged 5–13 years was estimated to provide an acceptable precision for the incidence estimation.

### Case definitions.

Definitions for suspected dengue, laboratory-confirmed and laboratory-probable dengue cases, and inapparent dengue infection are described in Table 1.

**Table 1:** Dengue case definitions

Case	Definition
Suspected symptomatic dengue	Febrile illness with a body temperature ≥ 38°C (by any route) measured on two consecutive days, with or without the presence of other dengue symptoms and without an obvious reason to suspect a condition other than dengue (based on a physician’s judgment).
Laboratory diagnosis of suspected dengue cases
Laboratory-confirmed dengue*	Positive DENV identification by serotype-specific quantitative real-time polymerase chain reaction on acute blood samples, or anti-DENV IgM seroconversion between acute and convalescent samples, or anti-DENV IgG “capture conversion”: negative IgG capture result on acute blood samples followed by a positive IgG capture result on convalescent blood samples.
Laboratory-probable dengue	Anti-DENV IgM or IgG positivity in at least 1 sample (acute or convalescent) and no evidence of viremia in acute samples and no evidence of anti-DENV IgM or IgG seroconversion between acute and convalescent samples.
Indeterminate dengue	Negative result for anti-DENV IgM and IgG antibodies in acute samples and no evidence of viremia in acute samples and convalescent samples not available.
Negative	Cases not eligible for the aforementioned categories, excluding “unknown” cases where no laboratory result was available, or the acute sample result was missing and the convalescent sample was negative.
Clinical severity of laboratory-confirmed symptomatic cases
Laboratory-confirmed symptomatic dengue case, mild	Laboratory-confirmed dengue case with a body temperature ≥ 38°C measured on two successive days and no criterion for moderate to severe dengue.
Laboratory-confirmed symptomatic dengue case, moderate to severe	Laboratory-confirmed dengue case with at least one criterion: increased vascular permeability documented by objective evidence, such as hemoconcentration ≥ 20% or the accumulation of a pleural effusion (documented by right lateral decubitus chest X-ray or ultrasound on the day of defervescence or no later than one day after defervescence; a pleural effusion index of > 4% was considered as evidence of plasma leakage) or ascites (documented by ultrasound greater than “trace” fluid); liver injury manifested as a maximum alanine aminotransferase or aspartate aminotransferase ≥ 125 units/liter; platelet count < 100,000 cells/mm^3^; respiratory insufficiency with oxygen saturation < 90% as assessed by minimum O_2_ saturation by room-air pulse oximetry and measured on at least two occasions at least 1 minute apart; gastrointestinal hemorrhage (documented hematemesis, melena, or hematochezia); moderate to severe hemorrhage involving other sites or tissues, for example, prolonged epistaxis (requiring pressure > 15 minutes to end blood flow), oral bleeding (intermittent bleeding from gums, lips, buccal mucosa, and posterior oropharynx), widespread ecchymoses (> 5 lesions larger than 3 cm), and menometrorrhagia; altered mental status, as evidenced by disturbance of consciousness (e.g., reduced clarity of awareness of the environment; inability to focus, sustain, or shift attention) and/or a change in cognition (e.g., memory impairment, disorientation, language disturbance, or development of a perceptual disturbance); and death plausibly related to dengue.
Inapparent dengue infection
Inapparent primary dengue	Occurrence of anti-DENV IgG seroconversion (using an indirect IgG ELISA) between two sequential sera samples obtained during scheduled visits 1 to 4. In this context, overt dengue illness was not suspected during the period in which seroconversion occurred.

DENV = dengue virus.

* To determine serologic status, IgM and IgG ELISA capture assays were used, where the IgG capture assay detects IgG antibodies characteristic of secondary dengue infections. Nota bene: IgG “capture conversion” only confirms that the IgG capture assay for this sample was negative according to the assay threshold.

### Laboratory assays.

The RT-qPCR assay for laboratory diagnosis of SDCs used a modified-assay protocol compared with the one previously described.^25^ The detection limits for the RT-qPCR assay were as follows: 6,500 genome equivalents per milliliter (Geq/mL) for DENV-1, 160,000 Geq/mL for DENV-2, 1,250 Geq/mL for DENV-3, and 33,000 Geq/mL for DENV-4.

In addition to RT-qPCR, serological assays were also used for the laboratory diagnosis of suspected dengue, based on DENV-specific IgG/IgM antibody concentrations, which were measured in serum samples using dengue IgM capture ELISA and dengue IgG capture ELISA (Panbio, Brisbane, Australia). The IgG capture ELISA is set to detect high IgG levels characteristic of secondary infections. As summarized in Table 1, serological confirmation required the dengue IgM capture ELISA or the IgG capture ELISA to be negative on the acute blood sample and positive on the convalescent blood sample. Participants who did not meet this criterion but had at least one sample testing positive with IgM capture ELISA or IgG capture ELISA were considered as probable cases.

Dengue virus IgG seroprevalence at yearly scheduled visits was established using a dengue IgG indirect ELISA (Panbio).

Confirmed and probable dengue cases were further investigated by a plaque reduction neutralization test (PRNT_50_) assay adapted from the WHO guidelines (exploratory objective).^26^ Dengue virus neutralizing antibody (Nab) titers were measured for DENV-1, DENV-2, DENV-3, and DENV-4 on the samples collected at the scheduled visits preceding and following the suspected dengue episodes; ≥ 4-fold rises in PRNT_50_ titers (reciprocal of the serum dilution that reduces the number of dengue viral focus forming units by ≥ 50% compared with the virus control) for at least one DENV serotype between both scheduled visits were considered as indicative of dengue exposure. Three-fold serial dilutions of serum tested in triplicate (starting at dilution 1:10 to 7,290) are incubated with a fixed amount of DENV serotype. The mixture is then added to a monolayer of preestablished Vero cells, and the replication of the virus is revealed by addition of an anti-*Flavivirus* monoclonal antibody (MAb 4G2) followed by a goat anti-mouse polyclonal antibody conjugated to horseradish peroxidase (HRP). The HRP activity is detected using precipitating tetramethylbenzidine substrate (True Blue™, SeraCare Life Sciences, Inc., Gaithersburg, MD) resulting in a coloration of the dengue-infected Vero cells. The viral plaques are counted, and the ratio between the number of plaques for each serum dilution and the number of plaques when no serum is added (virus control wells) is calculated. The serum Nab titer is reported as the reverse of the highest serum dilution reducing by 50% the number of viral plaques as compared with virus control without serum (end point dilution 50%, ED50).

### Statistical analyses.

The total cohort included all children enrolled in the study. Analyses were performed on children who met all the eligibility criteria and complied with the protocol-defined procedures.

The demographic and socioeconomic characteristics and the general medical history of the participants at enrollment were summarized. The distribution of visits with and without suspicion of dengue (stratified by case definition as described in Table 1) was summarized by calendar year and overall.

Dengue virus IgG seroprevalence with 95% confidence intervals (CIs) at enrollment, for each wave of enrollment, and by yellow fever vaccination status were tabulated overall and stratified by age. Seroprevalence was calculated as the number of participants who had previous dengue infection divided by the total number of participants with laboratory results available. An exploratory analysis of determinants of dengue IgG seropositivity at enrollment was performed. The potential determinants included demographic and socioeconomic characteristics (age, gender, family income, number of children, and adults in the household in relation to the number of rooms used for sleeping, access to water in the home, and type of health insurance) and other characteristics (school and the enrollment wave). Chi-squared tests and Wilcoxon–Mann–Whitney tests were used to investigate differences between seropositive and seronegative participants. Univariable and multivariable logistic regression analyses with backward variable selection were used to identify determinants of IgG seropositivity at enrollment. The incidence rates of symptomatic dengue and their 95% CIs were calculated as the total number of incident symptomatic dengue cases (first event per child) during the follow-up period per total time of follow-up for participants at risk of infection under surveillance. Rates were expressed as the number of cases per 1,000 child-years. Incidence rates for all symptomatic and laboratory-confirmed dengue cases were calculated for each calendar year, by age, and overall. Incidence rates were also calculated with 95% CIs for primary and secondary symptomatic infections. The incidence proportion of inapparent infections and its 95% CI was estimated for each season. For the incidence of secondary symptomatic dengue infections, only the first secondary symptomatic dengue infection for each participant was considered.

For laboratory-confirmed and laboratory-probable dengue episodes, the proportions of children with Nab titers ≥ 10 PRNT_50_ were computed with 95% CIs for the four DENV serotypes, for samples collected at the scheduled visit preceding the dengue episode, during the entire study and per semester. Geometric mean titers (GMTs) were computed with 95% CIs for each DENV serotype. Geometric means of the individual ratio (GMR) of Nab titers, corresponding to the scheduled visit sample following the suspected dengue episode over the one preceding the episode, were also computed with 95% CIs for each DENV serotype. These analyses were performed post hoc.

Clinical symptoms of dengue infection since onset were tabulated for SDCs. Some direct (hospitalization and length of hospitalization) and indirect (days off work from the child’s caregivers) resource utilization/impact indicators associated with symptomatic dengue infection were estimated and summarized using descriptive statistics, such as means, medians, and ranges. For each child, two potential caregivers were considered, referred to as primary and secondary caregivers.

Statistical analyses were performed using the Statistical Analysis Systems software version 9.2.

## RESULTS

### Study population.

The total cohort included 2,117 children (1,253 enrolled during wave 1, 394 during wave 2, and 470 during wave 3). During the first enrollment wave, issues with the informed consent were identified for 178 children (verification of the status of the LARs), of whom 92 withdrew from the study. One child was excluded from the analyses because of protocol violation.

**Figure 2.**
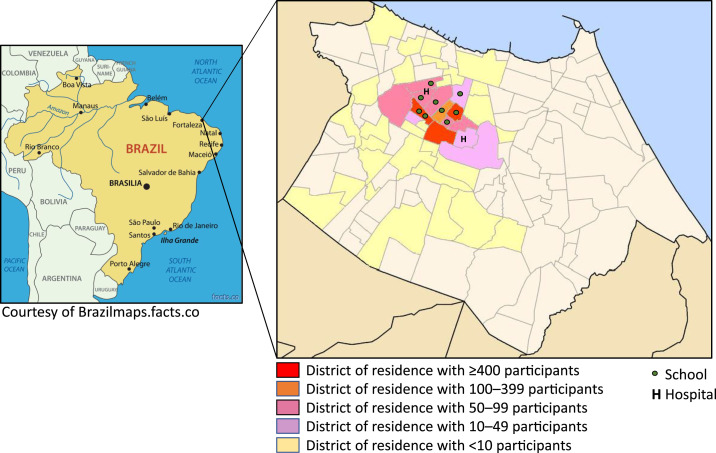
Geographical location and distribution of schools, study hospitals, and districts of residence of the study participants.

Children were recruited from nine schools (distant from each other by 1–3 km), with four schools recruiting 76.2% of the total cohort. Six schools participated to the first wave of enrollment and the nine schools participated to the second and third waves of enrollment. The number of children enrolled by school ranged from 21 to 600. The children resided in 44 districts of Fortaleza; 65.4% of them lived in three districts (Figure 2). The median age of the children at enrollment was 8.85 years, and 51.8% of them were girls. Table 2 provides a summary of the demographic and socioeconomic characteristics of all participants.

**Table 2:** Demographic and socioeconomic characteristics of study participants at enrollment (*N* = 2,116)

Parameter	Mean ± SD	*n* (%)
Age	8.9 ± 2.4	–
Gender		
Female	–	1,096 (51.8)
Male	–	1,020 (48.2)
Permanent residency in Fortaleza (all year through)		
Yes	–	2,072 (97.9)
No	–	44 (2.1)
Months per year the study participant lives in Fortaleza if not a permanent resident (*N* = 44)	5.8 ± 3.0	–
Monthly family income (average; R$)		
≤ 540	–	405 (19.1)
541−1,080	–	1,203 (56.9)
1,081−1,620	–	370 (17.5)
1,621−2,700	–	119 (5.6)
2,701−5,160	–	18 (0.8)
≥ 5,161	–	1 (0.1)
Health insurance		
Public only	–	2,003 (94.7)
Public and private	–	113 (5.3)
Household analysis		
The household visited by the family health program		
Yes	–	1,131 (53.5)
No	–	985 (46.6)
Number of household visits by the family health program per year (*N* = 1,129)	8.5 ± 7.2	–
Number of adults living in the household	2.4 ± 1.2	–
Number of children living in the household	2.7 ± 1.5	–
Number of rooms used for sleeping	2.0 ± 0.9	–
Number of people living in the household *over* number of rooms used for sleeping	2.8 ± 1.3	–
Running water from the public system inside home		
Yes	–	2,009 (94.9)
No	–	107 (5.1)
Frequency of water reaching home		
2–5 days per week and 3–12 hours per day	–	2 (0.1)
2–5 days per week and 13–24 hours per day	–	9 (0.5)
6–7 days per week and 3–12 hours per day	–	25 (1.2)
6–7 days per week and 13–24 hours per day	–	1,973 (98.2)
Missing	–	107 (−)
Other source of water supply		
Water tank/cistern/barrel	–	823 (77.8)
Well	–	122 (11.5)
Water tank/cistern/barrel and well	–	113 (10.7)
Missing	–	1,058 (−)
Type of home structure		
Masonry	–	2,100 (99.2)
Wooden	–	4 (0.2)
Other	–	12 (0.6)

*N* = number of children who met all the eligibility criteria and complied with the protocol-defined procedures; *n* (%) = number (percentage) of participants in a given category; R$ = Brazilian real. *N* = 2,116, if not specified otherwise.

Of 2,116 participants enrolled, 1,867 (88.2%) performed visit 2 (83.8%, 93.1%, and 95.7% of the participants enrolled in waves 1, 2, and 3, respectively). A redistribution of children between schools in 2012 explains why a substantial number of participants enrolled in wave 1 did not attend the second scheduled visit. The number of participants attending the third scheduled visit was 1,731 (81.8%) (77.6%, 83.2%, and 91.7% of the participants enrolled in waves 1, 2, and 3, respectively). Participation to a fourth scheduled visit only involved participants enrolled in waves 1 and 2, as the study duration was extended from 2 to 3 years. In total, 1,283 (68.7%) participants attended a fourth scheduled visit (968 participants enrolled in wave 1 and 315 enrolled in wave 2). The periods during which the yearly scheduled visits occurred for each wave of enrollment are displayed in Figure 1. The median time between the first and the second visit was 357, 155, and 189 days for enrollment waves 1, 2, and 3, respectively. The median time between the second and the third visit was 355, 356, and 335 days for enrollment waves 1, 2, and 3, respectively. The median time between the third and the fourth visit was 337 and 335 days for enrollment waves 1 and 2, respectively.

### Seroprevalence of DENV IgG antibodies at enrollment.

**Figure 3.**
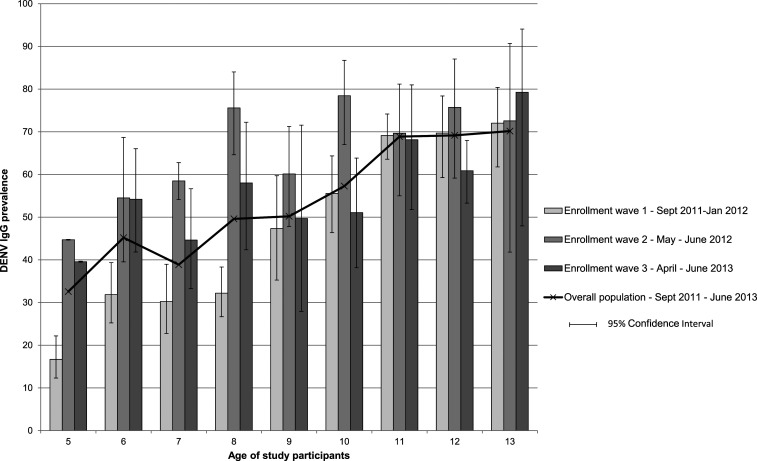
Prevalence of DENV IgG in study participants by age and enrollment wave (*N* = 1,059). DENV = dengue virus; *N* = number of study participants.

Of 2,104 children with available laboratory results, 54.1% (95% CI: 46.6, 61.4) were DENV IgG positive at enrollment. Among the 5- and 13-year-old children, 32.6% (95% CI: 26.1, 39.8) and 70.2% (55.8, 81.5) were DENV IgG positive, respectively (Figure 3).

The proportion of DENV IgG–positive children in the youngest age–groups was different across the different enrollment waves. The proportion of 5-year-olds who tested positive for IgG during enrollment waves 1, 2, and 3 was 16.7% (95% CI: 12.3, 22.2), 44.7% (44.6, 44.8), and 39.6% (39.5, 39.7), respectively. In 6-year-olds, these proportions were 31.9% (95% CI: 25.2, 39.4), 54.5% (39.5, 68.7), and 54.2% (41.8, 66.0), respectively (Figure 3). In the multivariate analysis, determinants of IgG seropositivity at enrollment were age in years (adjusted odds ratio [aOR] 1.29; 95% CI: 1.24, 1.35) and enrollment wave (aOR for wave 2: 2.23; 95% CI: 1.63, 3.05; aOR for wave 3: 1.59; 95% CI: 1.25, 2.03, compared with wave 1).

The number of children who were vaccinated against yellow fever was relatively small, with only 117 vaccinated children in total. In 5- to 9-year-olds with and without a history of yellow fever vaccination, the proportion of DENV IgG–positive children at enrollment was 61.6% (95% CI: 61.5, 61.6) and 43.1% (35.9, 50.7), respectively. In 10- to 13-year-old children, these proportions were 70.8% (95% CI: 57.2, 81.5) and 64.8% (58.3, 70.8), respectively.

### Symptomatic DENV infection.

**Figure 4.**
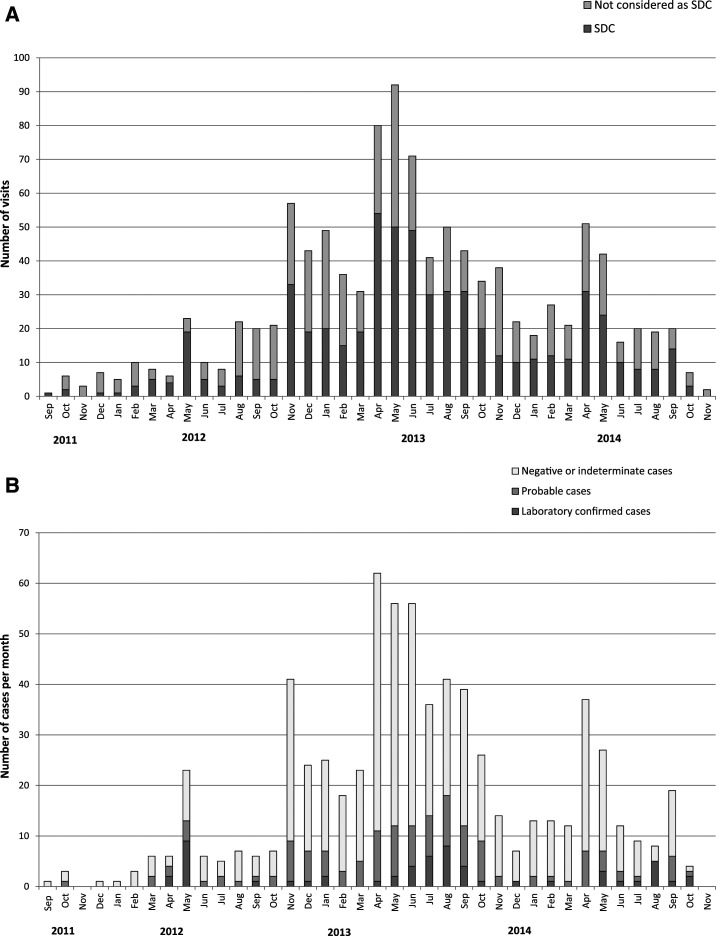
(**A**) Number of ad hoc medical visits by month and distribution of suspected dengue case (SDC) vs. non-SDC diagnoses following medical evaluation (*N* = 1,080). (**B**) Distribution of laboratory-confirmed, laboratory-probable, and negative dengue cases per month (*N* = 585).

Throughout the study, 1,080 visits for acute illness were carried out, of which 585 concluded in dengue suspicion following a physical examination (Figure 4A). Dengue suspicion was discarded in 495 visits, with an alternative diagnosis of upper respiratory tract infection (66.7% of visits without dengue suspicion), lower respiratory tract infection (4.4%), gastrointestinal infection (13.9%), urinary tract infection (0.4%), infectious disease other than dengue (5.5%), and noninfectious disease (9.7%). Most children with an episode of suspected dengue (92.5%) had not traveled in the 14 days preceding illness onset, whereas 5.3% had visited another location in the state of Ceará, 0.3% had traveled without specifying their destination, and 1.9% did not provide information regarding their recent travel history. Following the initial visit for suspected dengue, 11.1% of children returned for a medical visit for the same episode, with a median time between the visits of 3 days.

Among the 585 SDCs, 57 laboratory-confirmed dengue cases (of which 21 had a positive PCR result), 131 laboratory-probable dengue cases, and 340 cases negative for dengue infection were identified (Figure 4B, Table 3). In addition, 38 cases had an indeterminate diagnosis and 19 cases unknown results. The majority of confirmed and probable dengue cases (84.2% and 64.1%, respectively) occurred between April and September. The incidence of confirmed dengue was 11.0 (95% CI: 7.3, 14.7), 18.1 (10.4, 25.7), and 10.2 (0.7, 19.7) per 1,000 child-years, and of probable dengue 19.7 (9.7, 29.6), 42.3 (22.8, 61.8), and 16.1 (1.9, 30.4) per 1,000 child-years in 2012, 2013, and 2014, respectively.

**Table 3:** Detailed laboratory results for confirmed and probable dengue cases

Tests on acute and convalescent sample	DENV-neutralizing antibody profile at scheduled visits preceding and following the illness episode
Laboratory-confirmed dengue cases (*N* = 57)	
2 confirmed by RT-qPCR (14 DENV-4 and 7 DENV-1)	20 tested and 20 evocative of dengue exposure
7 confirmed by RT-qPCR and IgM seroconversion and IgG “capture conversion”	
3 confirmed by RT-qPCR and IgM seroconversion only	
5 confirmed by RT-qPCR and IgG “capture conversion” only	
6 confirmed by RT-qPCR only	
Serological confirmation	35 tested and 15 evocative of dengue exposure
33 cases with a negative RT-qPCR result and confirmed by IgM seroconversion between the acute and convalescent sample	
3 cases with a negative RT-qPCR result confirmed by IgG capture “positivation” only (negative on acute sample and positive on convalescent sample)	
Probable dengue cases (*N* = 131)	
36 considered as probable cases based on both IgM and IgG capture results	106 tested and 24 evocative of dengue exposure
53 considered as probable cases based on the IgM results only	
42 considered as probable cases based on the IgG capture results only	

DENV = dengue virus; *N* = number of children; RT-qPCR = quantitative real-time polymerase chain reaction.

For 20 of 21 cases confirmed by RT-qPCR, samples collected at the scheduled visits preceding and following the episode of dengue were tested by PRNT_50_. All these 20 cases had a dengue Nab profile evocative of dengue infection (≥ 4-fold rise in titers for at least one serotype between both scheduled visits). Of 36 cases confirmed by serology, 35 had scheduled visits samples tested by PRNT_50_; 15 had a Nab profile evocative of dengue infection. Finally, among 131 probable cases, 106 had samples tested by PRNT_50_ and 24 had a Nab profile evocative of dengue exposure. When considering only cases with an antibody profile corroborating dengue exposure, the incidence of dengue was 13.2 (95% CI: 6.6, 19.9), 18.7 (5.3, 32.2), and 8.4 (2.4, 19.2) per 1,000 child-years in 2012, 2013, and 2014, respectively.

At the scheduled visit preceding a dengue episode, 23/57 (41.1%) confirmed and 20/131 (15.5%) probable dengue cases had undetectable DENV IgG antibodies and were identified as primary cases. The yearly incidences were 11.2, 9.2, and 8.4 per 1,000 child-years for primary confirmed dengue cases and 10.9, 17.5, and 11.4 per 1,000 child-years for secondary confirmed dengue cases in 2012, 2013, and 2014, respectively.

Among the 21 symptomatic dengue infections confirmed by PCR, 14 children were infected by DENV-4 (in 2012 and 2013) and seven cases by DENV-1 (all in 2014).

The symptoms at onset and initial visit are described in the Supplemental Material.

### Seroconversions between scheduled visits and inapparent primary DENV infection (seroconversion of DENV-naive children between two scheduled visits without record of dengue suspicion).

Of 953 children who were dengue naive (DENV IgG negative) at enrollment and had at least one subsequent scheduled visit with blood collection, 313 (32.8%) became positive for DENV IgG (seroconversion), reflecting the proportion of primary dengue infections (symptomatic and inapparent) during the course of the study. In 2012, the proportion of seroconversions was 32.8% and 2% for children enrolled in wave 1 (seroconversions occurring between September 2011 and December 2012) and wave 2 (seroconversions occurring between May 2012 and December 2012), respectively. For the period going from October 2012 to December 2013, it was 13%, 10.5%, and 12% for children enrolled in waves 1, 2, and 3, respectively. Finally, for the period between October 2013 and January 2015, it was 4.7%, 5%, and 8.2% for children enrolled in waves 1, 2, and 3, respectively.

The proportion of dengue-naive children who developed a primary inapparent dengue infection during follow-up was 20.3% (95% CI: 13.6, 29.1), 8.7% (6.9, 11.0), and 5.1% (4.4, 6.0) in 2012, 2013, and 2014, respectively. In 2012, proportions of primary inapparent dengue infections were 30.3% (95% CI: 24.8, 36.5) and 3.1% (1.0, 9.8) for children enrolled in waves 1 and 2, respectively. In 2013, this proportion was 7.7% (95% CI: 6.1, 9.7) for children enrolled in waves 1 and 2, and 9.9% (7.4, 13.1) for children enrolled in wave 3.

### Dengue virus Nab profiles.

Among children with a confirmed or probable dengue episode who had available samples for Nab testing, 18.5% had no DENV Nab, 7.6% had DENV Nab against one serotype, 2.5% against two serotypes, 4.5% against three serotypes, and 66.9% against four serotypes at the scheduled visit preceding the episode. The observed GMRs of DENV Nab GMTs measured at the scheduled visit following the dengue episode over the one preceding the episode were higher for DENV-4 than for the other DENV serotypes for laboratory-confirmed dengue cases (Table 4).

**Table 4:** Geometric mean titers and GMRs for samples collected at the scheduled visits following over-preceding the suspected dengue episodes for DENV Nab titers (laboratory-confirmed and probable dengue episodes)

Antibody	*N*	GMT before episode	GMT after episode	GMR (95% CI)
Laboratory-confirmed episodes				
DENV-1	54	48.5	283.5	5.8 (3.0, 11.3)
DENV-2	54	33.8	193.9	5.7 (3.3, 9.9)
DENV-3	54	49.7	220.7	4.4 (2.6, 7.5)
DENV-4	54	37.9	441.7	11.7 (6.1, 22.4)
Laboratory-probable episodes				
DENV-1	106	365.8	538.2	1.5 (1.1, 2.0)
DENV-2	105	261.8	370.7	1.4 (1.0, 2.0)
DENV-3	105	483.0	552.5	1.1 (0.9, 1.4)
DENV-4	105	238.6	364.5	1.5 (1.1, 2.1)
Laboratory-confirmed and laboratory-probable episodes				
DENV-1	154	172.5	413.9	2.4 (1.7, 3.3)
DENV-2	153	119.8	281.2	2.3 (1.7, 3.2)
DENV-3	153	207.5	384.5	1.9 (1.4, 2.4)
DENV-4	153	120.4	380.1	3.2 (2.2, 4.5)

DENV = dengue virus; GMR = geometric mean ratio; GMT = geometric mean antibody titer; *N* = number of dengue episodes with available laboratory results at the two considered time-points; Nab = neutralizing antibody.

When comparing the DENV IgG indirect ELISA assay with PRNT_50_ on the subset of 359 scheduled visit samples tested with both assays, the sensitivity of the DENV IgG indirect ELISA assay was 97%, the specificity 95%, the positive predictive value 99%, and the negative predictive value 85%.

### Dengue disease severity.

None of the confirmed dengue cases met the prespecified study criteria for moderate to severe dengue (Table 1). Other symptoms considered as moderate to severe, which were not part of the prespecified criteria, were recorded for three children (abdominal pain; intense nausea, vomiting, and unrest; and irritating dry cough). Among the probable dengue cases, five children reported symptoms indicating a moderate to severe dengue episode (gastrointestinal hemorrhage [1], intense nausea and vomiting [3] accompanied by profuse cold sweats [1], and fainting [1]).

During the study, two children were hospitalized for a confirmed dengue infection. One child with a diagnosis of moderate gastritis was hospitalized for 19 days in 2012, whereas the other child was hospitalized for 4 days in 2014. Both children were released from hospital with no further complications. None of the children with probable dengue infection were hospitalized.

Further information on the symptoms reported at illness onset and initial medical visits is available in Supplemental Table 1. There were no reports of dengue-related deaths throughout the study.

### Indicators of dengue illness impact.

Of the 57 children with confirmed symptomatic dengue, 86% missed school because of illness, with a median duration of 4 days. The primary and secondary caregivers of 21.1% and 7.0% of children with confirmed symptomatic dengue, respectively, missed work because of the illness of their child, with a median duration of 1.5 days.

## DISCUSSION

To our knowledge, this is the first large longitudinal pediatric cohort study assessing the burden of dengue disease in a school population in Brazil. Similar cohort studies have previously been conducted in other countries, such as Thailand and Nicaragua.^27,28^ A multicenter cohort study was also conducted in 3,000 children aged 9–16 years across 20 sites in Latin America, among which five were in Brazil.^9^

In our study, 57 laboratory-confirmed and 131 laboratory-probable dengue cases were identified in 5- to 13-year-old children. The incidence of laboratory-confirmed dengue cases was 11.0, 18.1, and 10.2 per 1,000 child-years, and the incidence of dengue cases with Nab profiles evocative of dengue exposure was 13.2, 18.7, and 8.4 per 1,000 child-years in 2012, 2013, and 2014, respectively. Our results were comparable with incidence rates in other endemic settings.^9,27,29^ In the previous multicenter study conducted in Latin America, the incidence of laboratory-confirmed dengue cases in Fortaleza was 1.61 and 6.56 per 1,000 children aged 9–16 years in 2010 and 2011, respectively.^9^ The estimated incidence of dengue based on the notification of cases to public health authorities in Fortaleza was 12.69, 3.28, and 1.53 per 1,000 children aged 5–9 years and 18.13, 4.17, and 2.56 per 1,000 children aged 10–17 years in 2012, 2013, and 2014, respectively (communication from the epidemiological surveillance unit of the Health Secretary of Fortaleza, Supplemental Table 2). In 2012, the incidence estimated in our study was similar to that estimated from the notifications. However, incidence rate estimates in 2013 and 2014 were around three to five times higher in our study than those reported to the Fortaleza Health Secretary in 2013 and 2014. This suggests that cases were more efficiently detected by active surveillance in our study than the case notification to public health authorities for the years 2013 and 2014, but not in 2012. In a previous cohort study conducted in schoolchildren in Nicaragua, the incidence of symptomatic disease (8.5 and 8.3 per 1,000 children in the 2001–2002 and 2002–2003 seasons) was also significantly higher than that reported to the Ministry of Health (0.8 and 0.3 per 1,000 children for the same periods), indicating that the study surveillance system was at least 10-fold more sensitive than the national dengue surveillance system.^30^ In another cohort study conducted between 2004 and 2008 in Nicaragua, on average, 21 times more dengue cases were identified compared with the number reported to the National Epidemiologic Surveillance program.^31^

Among laboratory-confirmed and laboratory-probable symptomatic cases, the proportion of primary dengue infection was 41.1% (23/57) and 15.5% (20/131), respectively. In our study, more than half of the children (54.1%) were positive for DENV IgG antibodies at enrollment. It may reflect not only previous exposure to dengue but also previous exposure to other flaviviruses (through infection or vaccination) because of the high cross-reactivity in the serology.^23^ We assumed that the high seropositivity rates at enrollment mostly reflected a previous dengue exposure as only a small proportion of children (< 6.0%) was vaccinated against yellow fever, and the study was conducted before the Zika virus epidemic which spread in Brazil in late 2014.^32^ Unsurprisingly, we found that age was a determinant of IgG seropositivity at enrollment. Children enrolled in waves 2 and 3 were also more likely than children enrolled in wave 1 to be seropositive for dengue IgG, suggesting high exposure to dengue between the first enrollment wave and the subsequent waves.

Among the 21 laboratory-confirmed symptomatic dengue cases with PCR results available, we detected only DENV-4 infections in 2012 and 2013, and only DENV-1 infections in 2014. Among laboratory-confirmed cases with a Nab profile available, the highest GMRs of DENV Nab titers between the samples taken before and after the dengue episode were observed for DENV-4, reflecting the high infection rate by this serotype. A previous study showed that DENV-4 had reemerged in the state of Roraima in Northern Brazil in 2010, after an absence of 28 years.^33^ In the state of Ceará, DENV-4 was detected in 2011, when it represented 0.9% of the samples.^34^ PCR testing on samples from acute cases as part of the virologic surveillance showed that DENV-4 was the predominant serotype circulating in the state of Ceará in 2012 and 2013, representing 98.5% and 96.7%, respectively, of tested samples. This proportion decreased to 43.2% in 2014, when DENV-1 and DENV-3 were found in 54.2% and 2.6% of all tested samples, respectively.^34^ Similarly, a prospective study in central Brazil showed that among 253 cases of laboratory-confirmed dengue that occurred between January 2012 and July 2013, DENV-4 was detected in the 55.6% of cases, DENV-1 in 37.4%, DENV-3 in 5.3%, and DENV-2 in 1.6% of cases.^35^ Most children were naive to DENV-4 at the beginning of the study, although there is evidence that some children already had detectable neutralizing antibodies against this serotype at the end of 2011.

During the course of the study, 32.8% of initially DENV IgG–negative children seroconverted. The highest proportion of seroconversion was observed for children enrolled in wave 1 during the 2012 season (32.8%). Such high rates of dengue infection have been reported occasionally in other settings. In Indonesia, a cohort study including 1,837 children aged 4–9 years reported an overall incidence of 29.2% for one or more DENV infections over the one-year study period.^36^ In Cebu City in the Philippines, the historical dengue infection rate among dengue-naive individuals was estimated to be at 11–22% per year.^37^

The remarkable difference in the proportion of seroconversions between the enrollment visit and the subsequent scheduled visit among children enrolled in wave 1 (32.8%) and those enrolled in wave 2 (2%) is most likely explained by the timing of visits and the seasonality of dengue transmission. For wave 1, seroconversions occurred between September 2011 and December 2012, whereas for wave 2, seroconversions occurred between May 2012 and December 2012. This suggests than dengue transmission intensity was the highest in the first part of 2012 (until May/June), which is consistent with the historical seasonality of the disease in Fortaleza and with the distribution of reported cases in the state of Ceará during 2012.^38^ This coincides with the introduction of DENV-4 in a population which had never been exposed to this serotype. Dengue transmission is known to be highly focal, and variations of incidence by district have been reported in Fortaleza.^39^ Although the school and home location may relate to the risk of exposure to infectious mosquito bites, it is unlikely that differential geographical exposures explain the difference in seroconversion between enrollment waves 1 and 2. The schools were not very distant from each other, and the schools participating to enrollment wave 1 also took part to enrollment wave 2.

In the following seasons, the proportion of seroconversions was notably lower, with an observed 10.5–13% in 2013 and 4.7–8.2% in 2014, which is in the range of the proportion of infections detected by many prospective cohort studies.^40,41^

The proportion of children with inapparent dengue infection (seroconversions among children without dengue suspicion) was particularly high in 2012 (20.3%), especially for children enrolled in wave 1 (30.3%), in comparison with the subsequent years (8.7% in 2013 and 5.1% in 2014). Although the incidence of laboratory-confirmed dengue was similar between study years, the higher incidence of inapparent dengue infection suggested higher dengue infection rates during the first months of 2012, during the large DENV-4 outbreak in Fortaleza. The higher proportion of children with inapparent infection compared with symptomatic dengue infection during the first months of the study can be explained by the underestimation of symptomatic dengue cases through the reporting of school absenteeism, which was not yet optimal.

Most dengue episodes observed in the study population were mild, with only two recorded dengue-related hospitalizations and no dengue-related deaths. At initial presentation, headache, gastrointestinal, and respiratory symptoms were reported by ≥ 50% of children with laboratory-confirmed dengue episodes and a DENV Nab profile evocative of dengue exposure. Similar clinical observations were made in other studies conducted in Brazil^42,43^ and in other endemic settings, such as Puerto Rico and Thailand.^44,45^

This study faced certain limitations. The case definitions for laboratory-confirmed and laboratory-probable dengue cases were based on the combination of virological (RT-qPCR) and serological test results (IgM and IgG capture ELISA). Indeed, RT-qPCR testing alone would not allow the diagnosis of children presenting for a medical consultation after more than 5 days following symptom onset. When examining Nab titers from sera collected at scheduled visits preceding and following a dengue episode, all cases confirmed by RT-qPCR had a profile evocative of dengue exposure. However, among the 36 cases considered as confirmed based on IgM and IgG capture ELISA results, only 15 had a Nab profile evocative of dengue exposure. This suggests that some children could have been falsely classified as dengue infected through ELISA, which is detecting the total antibody response. Of note, the ≥ 4-fold titer threshold used to detect variations in Nab titers with the PRNT assay in this study has also been used in other cohort studies as a marker of exposure to dengue infection to define asymptomatic/inapparent infections.^27,46^ In the comparison of Nab GMTs at the scheduled visits preceding and following dengue episodes, the highest ratio was observed for DENV-4 among laboratory-confirmed cases, which is coherent with the fact that DENV-4 was preponderantly circulating in 2012 and 2013. However, GMRs for DENV-2 and DENV-3 were also elevated. These serotypes have not been identified during the course of the study. In the state of Cearà, virological surveillance did not detect any case of DENV-2 and DENV-3 in 2012 and 2013; in 2014, a low level of DENV-2 infections was detected (2.6% of all tested isolates).^34^ Antibody responses to dengue infection in participants already exposed to flaviviruses are heterotypic as opposed to a primary infection, which elicits a monotypic antibody response. This explains why we also observed an increase in neutralizing antibodies for serotypes to which the study participants were most likely not exposed to.

The prevalence of DENV IgG was assessed at scheduled visits with a commercial ELISA. Although we did not perform a specific validation of this assay, we believe that it provided an acceptable indicator of past dengue exposure across study visits and seroconversions, as shown by the comparison between the DENV IgG indirect results and PRNT results in a subset of samples collected at scheduled visits.

Another limitation was the surveillance coordination of febrile illness via school absenteeism reporting that was not fully optimized at the start of the study and most likely led to under-detection of febrile illness and dengue cases in early 2012. Indeed, the proportion of children reporting febrile illness episodes that were not medically attended (as per self-report at the yearly scheduled visits) was high during the 2012 season (above 40.0%) and decreased during the subsequent years (18.0% and 17.0% in 2013 and 2014, respectively). In addition, we cannot fully exclude that the suspicion of dengue may have been erroneously discarded in some instances. About 67% of medically attended events not considered as suspected dengue included upper respiratory tract infection. In a study in Puerto Rico investigating the clinical and epidemiological aspects of febrile illness, symptoms of cough, rhinorrhea, and sore throat were reported in 26–37% of confirmed dengue cases.^47^ The proportion of patients with these respiratory symptoms, although not negligible, was however significantly lower than in infections due to influenza and other respiratory viruses. Similar proportions of respiratory symptoms were also reported in a study conducted in Brazil in dengue patients aged 12 years or older with fever lasting up to 3 days and without any evident focus of infection.^48^ In our study, the definition for a SDC was sensitive (requiring 2 days of fever), and only children presenting with symptoms suggesting an obvious other etiology were no longer considered as dengue suspected. The study protocol drew attention to the fact that along with classic dengue symptoms, dengue can also present as an acute respiratory illness. Therefore, we believe that the risk of erroneously discarding a suspicion of dengue was low.

In summary, there was a high dengue infection rate in 2012 during the DENV-4 outbreak in Fortaleza. The infection rate was lower in 2013 and 2014, with the continuous detection of DENV-4 in 2013 and recirculation of DENV-1 in 2014. More than half of the confirmed dengue cases were secondary infections, confirming the high endemicity of the disease in Fortaleza. Besides underlining the fluctuating nature of serotype-specific dengue burden over time in this region, our results also confirmed that cases were generally more efficiently detected by active surveillance than by the case notification to public health authorities. Most disease surveillance and notification systems are affected by a degree of underestimation, which hinders the estimation of the “true” disease incidence.^49^ It results from a failure to ascertain all cases, as not all cases seek health care or as some cases may not be correctly diagnosed and from the failure to adequately report cases that have sought medical advice and were correctly diagnosed. A laboratory-enhanced dengue sentinel surveillance in defined areas, with the participation of most outpatient clinics and hospitals, where dengue PCR diagnosis would be systematically performed for patients with undifferentiated febrile illness, could help improving the detection of cases and the assessment of disease incidence across various age-groups. The study also brought insight on the age-specific seroprevalence of dengue, which is an important parameter for the design of vaccine trials and vaccination policy. Seroprevalence estimates in children may differ from 1 year to another, as a consequence of outbreaks. This factor should be tailored in countries/regions that plan to determine their vaccination policy and the age-specific recommendation based on serological surveys.

## Supplemental material and tables

Supplemental materials
