# Supplementary material for: Dengue Infection in Children in Fortaleza, Brazil: A 3-Year School-Based Prospective Cohort Study
Source: Am J Trop Med Hyg. 2020 Apr 27;103(1):100–11. doi: 10.4269/ajtmh.19-0521 (PMC7356456; doi:10.4269/ajtmh.19-0521)
Supplement: Supplementary file 1 [file tpmd190521.SD1.pdf]

SUPPLEMENTARY MATERIAL

Symptoms at onset and initial visit

The median time between fever onset (when fever was the first symptom) and the *ad-hoc* visit was 2 days (range 0–11 days) for all suspected dengue cases (N = 401). When symptoms other than fever were considered to mark the onset of illness, the median time between the onset of this symptom and the visit was 2 days (range 0–28 days).

The reported clinical symptoms representative of the initial visit are described in **Supplementary Table 1** for the following subgroups: laboratory-confirmed cases based on virological and serological criteria, laboratory-confirmed cases (virological and serological criteria) coupled with a Nab profile evocative of dengue exposure, laboratory-confirmed and laboratory- probable cases with a Nab profile evocative of dengue exposure, and cases considered negative for acute dengue infection.

The prevalence of symptoms was compared between children who had no laboratory evidence of dengue infection (negatives), and children confirmed as dengue-infected by RT-qPCR or IgM seroconversion (**Supplementary Table 1**).

Supplementary Table 1. Clinical presentations of study participants at their initial medical visit for suspected dengue, stratified by dengue case category.

	All laboratory-confirmed episodes (N = 57)	Confirmed episodes* (N = 35)	Confirmed and probable episodes† (N = 59)	Negatives (N = 340)
Characteristics	n (%)	n (%)	n (%)	n (%)
Headache	43 (75.4)	28 (80.0)	47 (79.7)	260 (76.5)
Retroorbital pain	22 (38.6)	13 (37.1)	20 (33.9)	70 (20.6)
Myalgia	29 (50.9)	16 (45.7)	22 (37.3)	120 (35.3)
Join pain	10 (17.5)	5 (14.3)	8 (13.6)	28 (8.2)
Chills	7 (12.3)	3 (8.6)	5 (8.5)	29 (8.5)
Rash	3 (5.3)	2 (5.7)	3 (5.1)	7 (2.1)
Itching	8 (14.0)	7 (20.0)	10 (17.0)	4 (1.2)
At least one digestive sign among first symptoms	37 (64.9)	23 (65.7)	37 (62.7)	209 (61.5)
Abdominal pain	16 (28.1)	8 (22.9)	14 (23.7)	123 (36.2)
Nausea or vomiting	28 (49.1)	18 (51.4)	28 (47.5)	148 (43.5)
Diarrhea	9 (15.8)	4 (11.4)	7 (11.9)	77 (22.7)
At least one respiratory sign among first symptoms	31 (54.4)	15 (42.9)	29 (49.2)	193 (56.7)
Cough	20 (35.1)	8 (22.9)	16 (27.1)	129 (37.9)
Nasal congestion	9 (15.8)	5 (14.3)	8 (13.6)	90 (26.5)
Sore throat	23 (40.4)	8 (22.9)	15(25.4)	93 (27.4)
Dyspnea	– (–)	– (–)	2 (3.4)	13 (3.8)
At least one hemorrhagic sign among first symptoms	4 (7.0)	2 (5.7)	4(6.8)	10 (2.9)
Petechia	3 (5.3)	2 (5.7)	2(3.4)	3 (0.9)
Purpura/ecchymosis	– (–)	– (–)	– (–)	1 (0.3)
Hematemesis	1 (1.8)	– (–)	– (–)	– (–)
Melena/hematochezia	– (–)	– (–)	1 (1.7)	1 (0.3)
Gingival bleeding	– (–)	– (–)	1 (1.7)	– (–)
Epistaxis	– (–)	– (–)	– (–)	3 (0.9)
Urinary tract bleeding	– (–)	– (–)	– (–)	1 (0.3)
Unusual vaginal bleeding	– (–)	– (–)	– (–)	1 (0.3)
At least one other sign among first symptoms	33 (57.9)	20 (57.1)	34 (57.6)	184 (54.1)
Pallor or cool skin	2 (3.5)	1 (2.9)	1 (1.7)	6 (1.8)
Conjunctivitis	2 (3.5)	2 (5.7)	2 (3.4)	8 (2.4)
Lethargy or restlessness	3 (5.3)	2 (5.7)	2 (3.4)	2 (0.6)
Dizziness	11 (19.3)	7 (20.0)	8 (13.6)	48 (14.1)
Thoracic pain	– (–)	– (–)	– (–)	8 (2.4)
Other	28 (49.1)	17 (48.6)	30 (50.9)	160 (47.1)

N, number of ad-hoc visits with suspicion of dengue; n/%, number/percentage of ad-hoc visits with suspicion of dengue in a given category; Nabs, neutralizing antibodies. *Laboratory-confirmed episode with a profile of Nabs evocative of dengue exposure. †Laboratory-confirmed and laboratory-probable episode with a profile of Nabs evocative of dengue exposure.

Supplementary Table 2. Epidemiological data provided by the Cearà epidemiology surveillance on the incidence of dengue disease monitored by the public health surveillance system (through mandatory reporting) for (A) the entire pediatric population of Fortaleza and (B) the pediatric population living in the districts where most of the children followed during the present study live (Amadeu Furtado, Bela Vista,Bom Futuro, Damas, Jardim América, Montese, Parreão, Parquelandia, Pici [Parque Universitario], Rodolfo Teófilo)..

A. Entire pediatric population of Fortaleza

Period	1 Jan–31 Dec 2012		1 Jan–31 Dec 2013		1 Jan–31 Dec 2014	
Age group	5–9 year	10–17 year	5–9 year	10–17 year	5–9 year	10–17 year
Number of confirmed dengue cases	2281	6354	601	1493	283	923
- number of DWC/DWS cases	DWC: 2	DWC: 9	DWC: 2	DWC: 7	DWS: 10	DWS: 33
- number of DHF/SD cases	DHF: 1	DHF: 9	DHF: 0	DHF: 10	SD: 1	SD: 4
- number of dengue-related deaths	0	0	0	1	1	3
Size of pediatric population	179,686	350,478	183,383	357,723	184,820	360,523
Incidence of confirmed dengue cases/1,000 children	12.694	18.130	3.277	4.174	1.531	2.560

DWC, dengue cases with complications; DHF, dengue hemorrhagic fever; DWS, dengue cases with warning signs, SD, severe dengue.

B. Pediatric population living in selected districts of Fortaleza

Period	1 Jan–31 Dec 2012		1 Jan–31 Dec 2013		1 Jan–31 Dec 2014	
Age group	5–9 year	10–17 year	5–9 year	10–17 year	5–9 year	10–17 year
Number of confirmed dengue cases reported among residents of Fortaleza for the age group	207	551	57	135	17	70
- number of DWC/DWS cases	DWC: 0	DWC: 2	DWC: 0	DWC: 0	DWS: 0	DWS: 0
- number of DHF/SD cases	DHF: 0	DHF: 1	DHF: 0	DHF: 0	SD: 0	SD: 0
- number of dengue-related deaths	0	0	0	0	0	0
Size of pediatric population residing in selected districts for the age group	10,754	21,607	10,977	22,049	11,063	22,228
Incidence of confirmed dengue cases /1,000 children	19.249	25.501	5.193	6.123	1.537	3.149

DWC, dengue cases with complications; DHF, dengue hemorrhagic fever; DWS, dengue cases with warning signs, SD, severe dengue.
